# Re-establishing Apoptosis Competence in Bone Associated Cancers via Communicative Reprogramming Induced Through Notch Signaling Inhibition

**DOI:** 10.3389/fphar.2019.00145

**Published:** 2019-02-27

**Authors:** Michela Colombo, Natalia Platonova, Domenica Giannandrea, Maria Teresa Palano, Andrea Basile, Raffaella Chiaramonte

**Affiliations:** ^1^Department of Health Sciences, University of Milan, Milan, Italy; ^2^Department of Oncology and Hemato-Oncology, University of Milan, Milan, Italy

**Keywords:** Notch, Jagged, Dll, apoptosis, drug resistance, metabolism, stem cell, anakoinosis

## Abstract

Notch and its ligands on adjacent cells are key mediators of cellular communication during developmental choice in embryonic and adult tissues. This communication is frequently altered in the pathological interaction between cancer cells and healthy cells of the microenvironment due to the aberrant expression of tumor derived Notch receptors or ligands, that results in homotypic or heterotypic Notch signaling activation in tumor cells or surrounding stromal cells. A deadly consequence of this pathological communication is pharmacological resistance that results in patient’s relapse. We will provide a survey of the role of Notch signaling in the bone marrow (BM), a microenvironment with a very high capacity to support several types of cancer, including primary cancers such as osteosarcoma or multiple myeloma and bone metastases from carcinomas. Moreover, in the BM niche several hematological malignancies maintain a reservoir of cancer stem cells, characterized by higher intrinsic drug resistance. Cell–cell communication in BM-tumor interaction triggers signaling pathways by direct contact and paracrine communication through soluble growth factors or extracellular vesicles, which can deliver specific molecules such as mRNAs, miRNAs, proteins, metabolites, etc. enabling tumor cells to reprogram the healthy cells of the microenvironment inducing them to support tumor growth. In this review we will explore how the dysregulated Notch activity contributes to tumor-mediated reprogramming of the BM niche and drug resistance, strengthening the rationale of a Notch-directed therapy to re-establish apoptosis competence in cancer.

## Introduction

Bone marrow (BM) is a supportive milieu for primary cancers including osteosarcoma (OS) or multiple myeloma (MM), derived from BM osteoblasts (OBLs) or resident plasma cells, but also hematological cancers that maintain a reservoir of cancer stem cells (CSCs) in the BM niche (Crews and Jamieson, 2012; Behrmann et al., 2018) and bone metastases from carcinomas (Mundy, 2002; Coleman, 2006). Bone localization is critical. Indeed, up to 85% of patients that die from breast, prostate, or lung cancer display bone involvement at autopsy (Mundy, 2002; Coleman, 2006).

Bone associated cancers share the propensity to colonize the BM and take advantage of this specialized niche that favors tumor growth and induces pharmacological resistance. Here, cancer cells establish a pathological communication with nearby cells, such as stromal and bone cells, inducing the release of pro-tumor factors and cytokines (Kan et al., 2016; Colombo et al., 2018).

**FIGURE 1:**
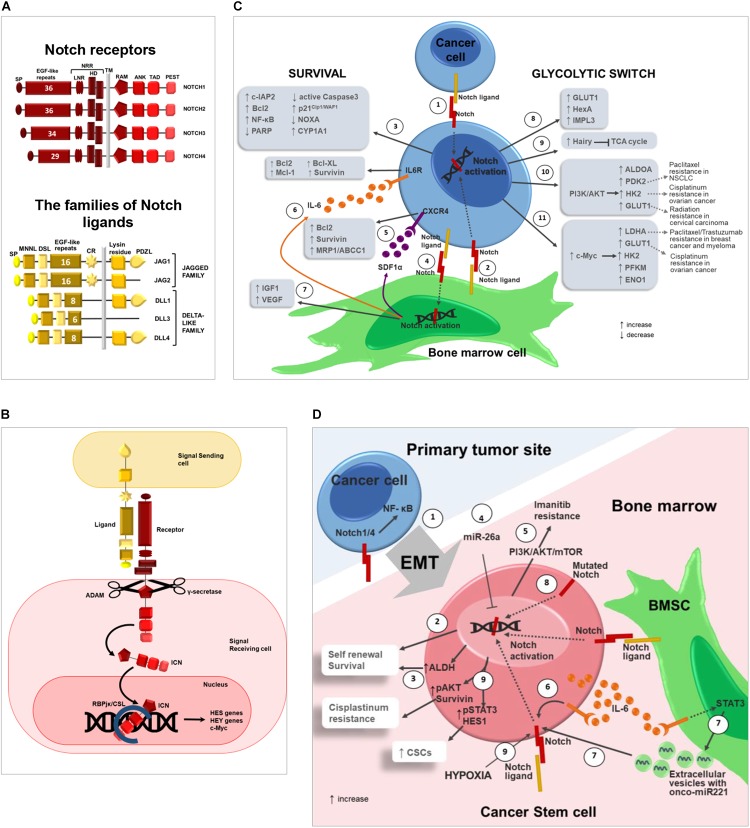
Notch pathway promotes drug resistance by regulating cancer cell survival, glycolytic switch and cancer stem cells. **(A)** Notch pathway can be triggered by the interaction of 4 receptors (Notch1-4) and 2 different classes of ligands, named Jagged (Jagged1-2) and Delta-like family (Dll1-3-4) (Platonova et al., 2015, 2017a,b). The following domains can be distinguished in Notch receptors: signal peptide (SP); epidermal growth factor(EGF)-like repeats; Negative Regulatory Region (NRR), composed by Lin-Notch repeats (LNR) and heterodimerization domain (HD); transmembrane domain (TM); RBJK associated module (RAM); ankyrin repeats (ANK); transactivation domain (TAD); proline(P),glutamic acid(E),serine(S) and threonine (T) domain (PEST). Jagged and Dll ligands are composed by: signal peptide (SP); Notch ligand N-terminal domain (MNNL); Delta/Serrate/LAG-2 domain (DSL); epidermal growth factor(EGF)-like repeats; cysteine rich region (CR); transmembrane domain (TM); Lysin residues; (PSD-95/Dlg/ZO-1)–ligand motif (PDZL) (Platonova et al., 2017a,b). **(B)** Canonical Notch signaling: Notch activation is triggered by ligand engagement which enables two consecutive proteolytic cleavages performed by the ADAM metalloproteinase and the γ-secretase complex, that allow ICN to translocate into the nucleus where it binds the RBJK/CSL complex and activates the transcription of Notch target genes such as the *HES* (Kageyama et al., 2007), and *HEY* (Weber et al., 2014) family of genes, *c-Myc* (Sato et al., 2016) and other genes involved in proliferation, survival, differentiation and stemness. **(C)** Notch role in cancer cell drug resistance. Notch activation in cancer cell can occur through: (1) homotypic interaction with nearby cancer cells or (2) heterotypic interaction with BM cells (i.e., BMSC). (3) Notch ligands localized on the surface of BMSCs activate Notch signaling in tumor cells resulting in increased expression of anti-apoptotic proteins including c-IAP2, Bcl-2, NF-κB and decreased expression of PARP and active Caspase3 (Nwabo Kamdje et al., 2011, 2012; Takam Kamga et al., 2016) with the subsequent development of chemoresistance mechanisms in different tumors as CLL (Nwabo Kamdje et al., 2012), B-ALL (Nwabo Kamdje et al., 2011) and AML (Takam Kamga et al., 2016). Moreover, BMSC-derived Notch ligands may stimulate the expression of p21^Cip1/WAF1^ and CYP1A1 and downregulate pro-apoptotic NOXA in cancer cells via Notch signaling regulating the development of drug resistance in MM cells (Nefedova et al., 2004, 2008; Xu et al., 2012a,b). (4) On the other hand, also cancer cells may activate Notch signaling in BM cells such as BMSCs, that in turn secrete the following pro-tumoral soluble factors: (5) SDF1α promotes and upregulates Bcl-2, Survivin and MRP1/ABCC1 in MM (Garavelli et al., 2017); (6) IL6 (Colombo et al., 2016) is reported to upregulate anti-apoptotic and pro-survival proteins in tumor cells including Bcl-2, Mcl-1, Bcl-X_L_, and Survivin (Catlett-Falcone et al., 1999; Shain et al., 2009; Ara and Declerck, 2010); (7) IGF1 and VEGF can contribute to induce drug resistance in hematological and solid tumors (Dias et al., 2002; Belcheva et al., 2004; Zhang et al., 2006; Kuhn et al., 2012; Hua et al., 2014; Nusrat et al., 2016; Bendardaf et al., 2017). Notch pathway may influence tumor cell drug sensitivity also promoting the glycolytic switch by enhancing the expression of glucose transporters and glycolytic enzymes in cancer cells. (8) In *D. melanogaster* Notch signaling was found to regulate GLUT1, HexA and IMPL3 and (9) suppress TCA cycle via the upregulation of Hairy gene (Slaninova et al., 2016). (10) In breast cancer Notch signaling induces PI3K/AKT activation that leads to the upregulation of glycolytic enzymes such as ALDOA, PDK2, HK2, and GLUT1 (Landor et al., 2011). Notably, BM adipocytes enhance the expression of two acknowledged Notch downstream effectors, i.e., HK2 and GLUT1, in prostate cancer cells (Diedrich et al., 2016). (11) In CLL, Notch pathway activation induces c-Myc upregulation and the subsequent increased expression of LDHA, GLUT1, HK2, PFKM, and ENO1 (Dang et al., 2009). Finally, wherever possible, for each Notch downstream effector involved in cancer cell glycolytic switch, it is reported the recognized outcome in drug and radiation resistance (Zhao et al., 2011; Huang et al., 2014; Lin et al., 2015; Sun et al., 2017; Zhang et al., 2018). **(D)** Notch in cancer stem cells: (1) Notch signaling promotes cancer cell EMT, which is closely associated to stemness. For instance, Notch1 and Notch4 expression in prostate cancer cells promotes EMT via NF-κB activation. EMT also enables cancer cell dissemination throughout the body including BM (Shibue and Weinberg, 2017; Zhang L. et al., 2017; Lin et al., 2018). (2) At BM level, Notch pathway activation in CSCs can be mediated by homotypic or heterotypic interactions and positively regulates tumor cell self-renewal, resulting in the amplification of the CSC population characterized by intrinsic high pharmacological resistance. (3) In OS cell, Notch activation boosts the expression of ALDH (Mu et al., 2013), a CSC marker also associated with drug resistance due to its detoxifying activity (Honoki et al., 2010). (4) miR-26a inhibits self-renewal by down-regulating Jagged1/Notch signaling (Lu J. et al., 2017). (5) In CLL, Notch activation results in resistance to Imatinib mediated by PI3K/AKT/mTOR signaling (Aljedai et al., 2015). (6) In BM microenvironment, tumor-derived IL-6 promotes an autocrine upregulation of Notch3, that in turn supports CSC survival and self-renewal (Sansone et al., 2016). (7) Moreover, IL-6 triggers STAT3 signaling in BMSCs improving the secretion of extracellular vesicles carrying the onco-miR221; this, in turn, increases Notch3 expression in CSCs and hormonal therapy resistance in bone metastasis of luminal breast cancer (Sansone et al., 2017). (8) In NSCLC, activating mutations of Notch1 correlate with CSCs and poor prognosis in patients (Westhoff et al., 2009). (9) In NSCLC, hypoxia-induced Notch1 activation promotes CSC self-renewal via pSTAT3 and HES1 and *cis*-platinum resistance through the positive regulation of pAKT and Survivin (Zhang Y. et al., 2017).

Notch pathway mediates cell–cell communication during cell fate decisions involved in embryonic development or adult tissues renewal (Siebel and Lendahl, 2017). Notch pathway is composed of a family of Notch receptors, Notch1-4, and two families of ligands, Jagged1, 2 and Dll1,3,4 (Figure 1A) (Platonova et al., 2017a). As illustrated by Figure 1B, Notch ligands bind to their receptors on adjacent cells inducing the release of the activated intracellular portion of Notch (ICN). ICN translocates into the nucleus and binds the RBJK/CSL complex triggering the transcription of target genes involved in proliferation, survival, differentiation and stemness (Platonova et al., 2017a). These include the *HES* (Kageyama et al., 2007) and *HEY* (Weber et al., 2014) families of transcriptional repressor genes, *c-Myc* (Sato et al., 2016), *cyclin-D1* (Ronchini and Capobianco, 2001), *p21* (Rangarajan et al., 2001), genes of NF-κB pathway such as *RELB* and *NFKB2* (Vilimas et al., 2007), and other genes which regulate the biological functions altered in cancer.

Additionally, non-canonical Notch signaling can occur independently from RBJK/CSL and dependently or not from ligand interaction. This exerts its biological functions interacting with other key signaling pathways, including PI3K, AKT, mTORC2, Wnt, NFκB, YY1, and HIF-1α, at cytoplasmic and/or nuclear levels (MacKenzie et al., 2004; Sade et al., 2004; Perumalsamy et al., 2009; Ayaz and Osborne, 2014; Platonova et al., 2015).

Due to the key role of Notch signaling in cellular communication, it is not surprising that its deregulation favors the pathological communication between cancer cells and BM cells.

This review represents a survey on Notch signaling activity in tumor-mediated reprogramming of the BM niche with the final purpose to provide evidence that dysregulated Notch pathway members may be rational therapeutic targets to re-establish apoptosis competence in cancer cells.

Here, we will focus on the ability of the reprogrammed BM niche to increase tumor cell resilience to chemotherapeutic agents, exploring the three main ways of BM cells involvement: (1) increasing the anti-apoptotic background of cancer cells; (2) inducing the glycolytic switch of tumor cells; (3) increasing the amount of CSCs.

## Notch Promotes BM-Mediated Increase of Anti-Apoptotic Background in Cancer Cells

Canonical and non-canonical notch pathway plays an acknowledged role in the regulation of apoptosis (MacKenzie et al., 2004; Sade et al., 2004; Perumalsamy et al., 2009; Platonova et al., 2015). Aberrant Notch signaling triggers anti-apoptotic program and drug resistance in different types of tumors, such as T-ALL (Sade et al., 2004), B-ALL (Nwabo Kamdje et al., 2011), CLL (Nwabo Kamdje et al., 2012), AML (Takam Kamga et al., 2016), Hodgkin and anaplastic large cell lymphoma (Jundt et al., 2002), cervical cancer cells (Perumalsamy et al., 2009), breast cancer (Meurette et al., 2009), MM (Nefedova et al., 2008; Mirandola et al., 2013; Ding and Shen, 2015; Garavelli et al., 2017), colon cancer (Meng et al., 2009), OS (Ma et al., 2013; Pu et al., 2017).

Figure 1C illustrates that Notch may be activated in tumor cells, but also in BM stromal cells (BMSCs) stimulating them to promote drug resistance. Indeed, BMSCs express Notch ligands, i.e., Jagged1, Jagged2 and Dll1, Dll3, and receptors, i.e., Notch1 and Notch2 (Bertrand et al., 2000; Nefedova et al., 2004; Xu et al., 2012a,b; Jitschin et al., 2015; Colombo et al., 2016; Sato et al., 2016), and many lines of evidence indicate that BMSC-derived Notch ligands may trigger Notch signaling in tumor cells inducing drug resistance. Krampera’s group widely demonstrated that Notch signaling activated by BMSCs promotes cell survival and chemoresistance in lymphoid neoplasms such as CLL [resistance to fludarabine, cyclophosphamide, bendamustine, prednisone, and hydrocortisone (Nwabo Kamdje et al., 2012)], B-ALL [resistance to hydrocortisone (Nwabo Kamdje et al., 2011)] and AML [resistance to cytarabine, idarubicin, and etoposide (Takam Kamga et al., 2016)] by increasing anti-apoptotic proteins including c-IAP2, Bcl-2, and NF-κB and reducing PARP and the active form of Caspase-3 (Nwabo Kamdje et al., 2011, 2012; Takam Kamga et al., 2016). Consistently, Notch depletion, induced by specific antibodies or γ-secretase inhibitor XII (GSI XII), reverted the protective effect of BMSCs (Nwabo Kamdje et al., 2011, 2012; Takam Kamga et al., 2016).

Notch signaling mediates also the pathological communications between BMSCs and MM cells promoting tumor cell survival and development of drug resistance (Colombo et al., 2013). MM cells accumulate in the BM, where they establish complex interactions with the surrounding healthy cells stimulating the release of anti-apoptotic factors relevant in drug resistance (Colombo et al., 2015a).

In the myelomatous BM, Notch signaling may be activated by a bilateral communication both in MM cells and in BM cells. Indeed, as reported for other hematological malignancies, BMSC-derived Notch ligands may activate Notch signaling by triggering Notch1 and Notch2 in myeloma cell (Colombo et al., 2013; Muguruma et al., 2017). BMSC-mediated Notch signaling activation has different outcomes, including the upregulation of p21^Cip1/WAF1^ and the downregulation of the pro-apoptotic protein NOXA in a p53-independent way (Nefedova et al., 2004, 2008). These, in turn, result in cell cycle slowdown and apoptosis resistance that protects tumor cells from apoptosis induced by chemotherapeutic drugs, such as doxorubicin, melphalan and mitoxantrone (Nefedova et al., 2004; Muguruma et al., 2017). Consistently, GSI-mediated inhibition of Notch signaling in MM cells significantly improved the response of MM cells to standard chemotherapy. In accordance, Xu et al. (2012a) demonstrated that BMSC-derived Dll1 activates Notch pathway in MM cells determining bortezomib resistance by upregulating CYP1A1, a member of the cytochrome P450 family involved in drug metabolism. In line with these data, the combined treatment of the syngeneic 5T33MM murine model with bortezomib and GSI resulted in increased bortezomib sensitivity and overall survival (Xu et al., 2012a).

In MM, the direction of the pathological communication may also be from tumor cells to BMSCs. Indeed, MM cells are reported to express Jagged ligands since the earlier stages. Jagged2 expression is already detectable in the benign form of MGUS and increases with disease progression (Houde et al., 2004), while Jagged1 increases during the progression from MGUS to MM (Skrtic et al., 2010). We used human MM cell lines, primary MM patients’ cells and a zebrafish MM model to show that MM cell-derived Jagged1 and 2 are pivotal to promote tumor cell ability to reprogram the nearby BM niche, and specifically to trigger BMSCs to protect MM cells from apoptosis induced by bortezomib, lenalidomide, and melphalan (Garavelli et al., 2017). The outcome of Notch signaling activation in BMSCs is the increased secretion of key cytokines, among which IL-6, IGF1, SDF1α, and VEGF (Houde et al., 2004; Colombo et al., 2014, 2016; Garavelli et al., 2017). These soluble molecules contribute to create a microenvironment favorable to tumor growth by regulating key biological processes such as cell survival and resistance to cytotoxic chemotherapy. Indeed, we showed that SDF1α, released by BMSCs upon MM-derived Jagged stimulation, determines MM cell resistance to the above reported drugs by promoting the expression of Bcl2, survivin and the multidrug resistance-associated protein 1 (MRP1/ABCC1) (Garavelli et al., 2017). IL-6 is widely involved in drug resistance induced by microenvironment. In MM cells IL-6 activates STAT3 signaling and increases the expression of antiapoptotic genes, such as Bcl2, Bcl-X_L_, Mcl-1, and survivin, commonly associated with chemoresistance (Catlett-Falcone et al., 1999; Shain et al., 2009; Ara and Declerck, 2010). IGF1 has been reported to promote MM cell resistance to bortezomib (Kuhn et al., 2012).

Although there is still no evidence concerning a role of VEGF signaling in MM drug resistance, it confers chemoresistance in several types of solid and hematological tumors (Dias et al., 2002; Belcheva et al., 2004; Zhang et al., 2006; Hua et al., 2014; Nusrat et al., 2016; Bendardaf et al., 2017) and involves anti-apoptotic effectors that play a role also in MM-associated drug resistance such a as Bcl2 and survivin (Gerber et al., 1998; Tran et al., 2002).

Similarly to MM cells, also bone metastatic breast cancer cells expressing high levels of Jagged1 activate Notch signaling in OBLs, thus stimulating the secretion of IL-6 that, in turn, favors tumor growth and chemoresistance (Sethi et al., 2011). On the other side, although the recognized role played by Notch signaling in OS pathogenesis (McManus et al., 2014; Tao et al., 2014) and multidrug resistance (Ma et al., 2013; Pu et al., 2017), it has not been established yet whether the underlying mechanism involves exclusively a tumor cell autonomous activation of Notch mediated by the high Dll1 level or if it may include also an increased Notch activity in the surrounding cells. Indeed, Pu et al. (2017) found that miR-34a-5p promotes OS multi-chemoresistance via repression of Dll1, indicating that targeting miR-34a-5p and Dll1 may provide a valuable strategy to overcome OS chemoresistance.

## Bone Marrow May Promote Drug Resistance by Activating the Glycolytic Switch in Tumor Cells Via Notch Signaling

Cancer cells may promote survival and proliferation by changing their metabolism, most frequently by increasing the glucose intake and consumption. In mammals, glucose is used for ATP production through glycolysis, TCA cycle and the OXPHOS in mitochondria. An aberrant feature of cancer cells is a significant increase of glucose uptake mainly exploited through glycolysis even in the presence of oxygen and intact mitochondria (Lu et al., 2015). This phenomenon is known as Warburg effect and recent studies indicate that it influences tumor cell drug sensitivity by enhancing drug efflux, DNA damage repair, survival and autophagy (Koppenol et al., 2011). Thereby, the onset of drug resistance is frequently associated with the upregulation of glycolytic key players including the glucose transporters and glycolytic enzymes (Butler et al., 2013). For instance, the upregulation of the glucose transporter 1 (GLUT1) is correlated with radiation resistance and poor prognosis in cervical squamous cell carcinoma patients (Huang et al., 2014; Lin et al., 2015). The increased expression of pyruvate kinase muscle isozyme 2 (PKM2) has been linked with resistance to Epirubicin and 5-fluorouracil in breast cancer patients (Lin et al., 2015), while its silencing potentiates the effects of oxaliplatin in colorectal cancer cells (Lu W.Q. et al., 2017). LDHA is associated with breast cancer resistance to paclitaxel/trastuzumab and myeloma relapse (Zhao et al., 2011); overexpression of hexokinase 2 (HK2) is involved in cisplatin resistance in ovarian cancer cells, by enhancing autophagy (Zhang et al., 2018) and the increased expression of PDK2 is linked to paclitaxel resistance in NSCLC (Sun et al., 2017).

The glycolytic switch in cancer cells is due to the activation of molecular pathways involved in the transcriptional regulation of several metabolic genes, among which the Notch signaling pathway (Figure 1C). Notch involvement was initially reported in *Drosophila melanogaster*, where it positively regulates genes encoding for *Glut1*, glycolytic enzyme hexokinase A (*Hex-A*), LDHA (*Ecdysone-inducible gene L3*, *Impl3*) and inhibits TCA cycle by upregulating the gene *Hairy*, which binds to the regulatory regions of TCA genes [Sdhb, l(1)G0255 and Kdn] suppressing their transcription (Slaninova et al., 2016).

Therefore, it is not surprising that Notch signaling dysregulation in cancer cells may also alter their metabolism. This effect has been mainly explored in breast cancer cells. Here, Notch activation may be induced by PEST mutations in Notch1-3 (Wang et al., 2015), high expression levels of Notch1, 3, 4, Jagged1, and Dll4 (Lamy et al., 2017; Kontomanolis et al., 2018), and BM-derived Notch ligands such as Jagged1 (Sethi et al., 2011; Zheng et al., 2017). *In vitro* and *in vivo* studies indicate that, in breast cancer, Notch signaling activation leads to increased glycolysis through the activation of the PI3K/AKT pathway, resulting in the upregulation of *GLUT1* expression and genes for rate-limiting glycolytic enzymes such as *HK2*, *ALDOA* and *PDK2*. Notch activation also induces GLUT1 translocation from cytoplasmic to membrane localization consistently with an increased glucose uptake due to glycolytic switch (Landor et al., 2011).

Recent evidence indicates that Notch pathway can participate also in the BMSC-induced metabolic switch. Indeed BMSC-derived Jagged1 plays an important role by favoring breast cancer bone metastasis formation and drug resistance (Sethi et al., 2011) and Landor et al. (2011) recently reported that Jagged1-mediated Notch activation in MCF7 cells promotes glucose consumption.

The role of BM stromal microenvironment in promoting the switch of malignant cells from mitochondrial respiration to glycolysis along with the acquisition of chemoresistance is recognized in different other tumor types. For instance, BMSCs induce the glycolytic switch in ALL (Frolova et al., 2012) and CLL cells (Vangapandu et al., 2017), promoting resistance to standard of care drugs including vincristine, methotrexate, and etoposide. Similarly, primary marrow fat cells and adipocyte cell lines trigger metabolic reprogramming of bone metastatic prostate cancer cells by enhancing the expression of PDK1, enolase 2 (ENO2), LDHA as well as HK2 and GLUT1 (Diedrich et al., 2016), already mentioned as Notch downstream effectors in breast cancer cells (Landor et al., 2011).

The role of Notch in energy metabolism has been explored also in CLL. Here, gain-of-function mutations of Notch1 [∼80% patients (Rosati et al., 2018)] stimulate a significant increase of glycolytic parameters (Jitschin et al., 2015). On the other hand, the activation of the four Notch isoforms expressed in CLL cells (Rosati et al., 2018) may be triggered also by BMSC-derived Notch ligands, i.e., Jagged1 and 2 and Dll3 (Jitschin et al., 2015). Consistently, BMSCs significantly increase the expression of glycolytic enzymes and glycolytic capacity. This effect is, at least in part, due to the promotion of Notch transcriptional activity on one of its most important targets, c-Myc that plays a recognized role in cancer cell energy metabolism by promoting the expression of *LDHA, GLUT1, HK2, PFKM* and *ENO1* (Dang et al., 2009). In accordance, GSI-mediated inhibition of Notch significantly increases CLL cell sensibility to used drugs, including Fludarabine and Ibrutinib (Jitschin et al., 2015; Secchiero et al., 2017).

The ability of Notch signaling to potentiate tumor chemosensitivity by interfering with another cellular metabolic pathway has been reported through *in vitro* and *in vivo* studies by Takam Kamga et al. (2018) who showed that Notch4 inhibition increases B-ALL sensitivity to the chemotherapeutic agent ara-C by upregulating the intracellular levels of ROS, which in turn, regulate mTOR, NF-κB, and ERK expression (Takam Kamga et al., 2018). Also, the same group showed the involvement of Notch3 and Notch4 in the pathological communication between B-ALL cells and the stromal microenvironment resulting in reduced drug sensitivity (Nwabo Kamdje et al., 2011).

The evident importance of the glycolytic switch in drug resistance development suggests that targeting cancer metabolism can be effective in restoring apoptosis competence in tumor cells. Growing evidence suggests that the metabolic reprogramming of cancer cells also depends on the interaction with the surrounding microenvironment and might be disrupted by inhibiting the pathological communication mediated by Notch signaling.

## Notch Activity Is Involved in the Maintenance and Expansion of Cancer Stem Cells

Cancer stem cells play a key role in the development of drug resistance, that crucially contributes to determine patient’s relapse and death (Phi et al., 2018). The characteristic resilience of CSCs to chemotherapeutic agents is due to different features. Indeed, (1) CSCs represent a reservoir of quiescent cells that undergo rare cellular division to maintain the bulk cell population, thereby resulting insensitive to anti-blastic treatment. (2) CSCs may efficiently extrude drugs through efflux pumps, including ABCB1, ABCG2, and ABCC1 (Moitra, 2015), (3) CSCs express high levels of detoxifying enzymes, such as ALDH (Nakahata et al., 2015), and (4) are characterized by a high anti-apoptotic background that hampers cell death upon treatment with pro-apoptotic drugs (Prieto-Vila et al., 2017; Phi et al., 2018).

These mechanisms of drug resistance can be promoted by the ability of different BM cellular players (i.e., BMSCs and OBLs) to activate pathways which allow CSCs to endure chemotherapy, survive as minimal residual disease and eventually prevail at relapse (Shiozawa and Taichman, 2012).

Here, we will explore Notch pathway ability to promote CSC self-renewal in several malignancies (Figure 1D), including T-ALL (Armstrong et al., 2009), CML (Aljedai et al., 2015), MM (Gao et al., 2016), OS (Mu et al., 2013), breast (Harrison et al., 2010a), prostate (Lin et al., 2018), and lung cancer (Hassan et al., 2013; Sosa Iglesias et al., 2018), and will report recent lines of evidence indicating that Notch signaling stimulates CSC crosstalk with the surrounding milieu resulting in a supportive feedback (Colombo et al., 2015b; Meurette and Mehlen, 2018).

In OS, Notch activation is associated with the expression of a CSCs marker, ALDH (Mu et al., 2013), whose detoxifying activity promotes cancer cell drug resistance (Honoki et al., 2010); while miR-26a inhibits CSC self-renewal ability and promotes chemosensitivity by suppressing Jagged1/Notch signaling (Lu J. et al., 2017).

Notch has been hypothesized to play a key role also in myeloma CSC maintenance (Colombo et al., 2015a). Indeed, Jagged2 expression in MM cell lines correlates with clonogenic ability and Notch-Fc chimeric molecules, uncoupling Jagged-Notch interaction, reducing colony formation *in vitro* and tumor formation in immunocompromised mice (Chiron et al., 2012). Concerning the microenvironment involvement, Notch signaling may be triggered also by BMSC-derived Dll1, resulting in increased MM cell clonogenic growth *in vitro* and tumor burden in 5T33MM syngeneic murine model (Xu et al., 2012b).

Also, leukemia stem cells (LSCs) rely on BM microenvironment to survive and propagate the bulk cell population (Povinelli et al., 2018). Aljedai et al. (2015) found a significant upregulation of *Notch1, Notch2* and the Notch-target gene *HES1* in the most primitive CD34^+^Thy^+^ subset of CML stem cells, suggesting that Notch pathway activation is critical for LSC population expansion. Interestingly, Notch activation results in imatinib resistance due to the activation of the compensatory PI3K-Akt/mTOR pathway, finally resulting in BCR/ABL-positive cells persistence that could be prevented by the combined inhibition of Notch and BCR-ABL (Aljedai et al., 2015).

In T-ALL, Notch1 activation promotes the growth and survival of the bulk cancer cell population along with LSCs self-renewal, as demonstrated by GSI inhibitory effect on cancer cell survival and the engraftment efficiency of primary human T-ALL cells in serial transplantation using immunocompromised mice (Belmonte et al., 2016). Moreover, T-ALL primary samples carrying mutated Notch1 showed a higher LSCs frequency and consequently an increased serial transplantation capacity *in vivo* respect to samples expressing the wild type form (Ma et al., 2012).

In solid tumors, Notch promotes CSCs self-renewal as well as EMT. This is of crucial importance, since EMT is closely associated to stemness and the activation of this program in malignant cells enables their conversion into CSCs, allowing them to form metastases and acquire drug resistance (Shibue and Weinberg, 2017). This connection is particularly evident in prostate cancer. Here, Notch4 activates NF-κB, boosting cancer cells growth and EMT (Lin et al., 2018); Notch1 promotes EMT, invasion and cell migration and EMT-like prostate cancer cells display a CSC phenotype (Zhang L. et al., 2017).

Notch receptors, including Notch1, 3, and 4, and the Jagged1 ligand support the expansion of CSCs and the development of drug resistance also in breast cancer. In HER2^+^ breast cancer, Notch signaling is associated to CSC resistance to the small molecule inhibitor of HER2, Lapatinib. This promotes Jagged1 expression in HER2^+^ CSCs, which, in turn, is associated with increased levels of Notch receptor expression and activity and CSCs enrichment *in vitro* and *in vivo*. Jagged1 predicts a poor overall survival (Shah et al., 2018) and is associated with bone metastasis (Zheng et al., 2017). Interestingly, in bone metastatic breast cancer, OBL-derived Jagged1 may induce drug resistance in tumor cells (Zheng et al., 2017). Other lines of evidence indicate that ESA^+^/CD44^+^/CD24^low^ breast CSCs showed high levels of Notch1 and Notch4, although Notch4 blockade was more effective in inhibiting tumor initiation *in vivo* (Harrison et al., 2010a,b).

In bone metastasis of luminal breast cancer, resistance to hormonal therapy is driven by a pool of self-renewing CD133^high^/ER^low^/IL6^high^ CSCs. High IL-6 levels stimulate Notch3 expression, that can replace the estrogen receptor signaling and promote CSCs survival and self-renewal (Sansone et al., 2016). In therapy-resistant stromal-tumor niches, IL-6/STAT3 signaling drives the expansion of BMSCs, that, in turn, secrete extracellular vesicles containing onco-miR221, able to induce hormonal therapy resistance through the generation of Notch3^high^/ER^low^/CD133^high^ CSCs (Sansone et al., 2017). Finally, Notch signaling positively regulates also NSCLC CSCs. Indeed, Notch1 mutations present in 10% of lung cancers, are associated with poor prognosis (Westhoff et al., 2009) and promote tumor initiation (Baumgart et al., 2015). Additionally, high levels of Notch1 may be induced in NSCLC CSCs by specific environmental conditions, such as tumor associated hypoxia (Zhang Y. et al., 2017). The hypoxic condition of BM and the availability of Notch ligands expressed by BM cells (Bertrand et al., 2000; Nefedova et al., 2004; Xu et al., 2012a; Jitschin et al., 2015; Colombo et al., 2016; Sato et al., 2016) suggest that Notch1 activation is not a limiting step in BM, and interestingly Notch1 activation promotes NSCLC CSC self-renewal via p-STAT3 and HES1 and induces resistance to *cis*-platinum treatment through the survival regulators p-AKT and survivin in a HES1-independent manner (Zhang Y. et al., 2017).

## Conclusion

We believe that the lines of evidence here reported and summarized in Table 1 clearly show that Notch signaling is instrumental in the pathological communication between tumor cells and BM leading to the reprogramming of surrounding microenvironment and the development of pharmacological resistance. Thus, targeting Notch pathway to prevent BM-mediated support promises to be effective in re-establishing apoptosis competence and overcoming drug resistance to eradicate the disease. Notably, although the important side effects of the currently used pan-Notch signaling inhibitors on small intestine, our survey points out that several tumors establish an aberrant communication with the surrounding microenvironment exploiting only one or few components of the pathway, thereby suggesting that a safer use is possible using the inhibitors specific for a single receptor or ligand recently developed (Wu et al., 2010; Platonova et al., 2017b).

**Table 1:** Summary of the mechanisms involved in Notch pathway ability to promote BM reprogramming and drug resistance.

Cancer type	Drug	Notch pathway members	Inhibitor tested	Cellular mechanism	Molecular mediators	Reference
B-CLL	Fludarabine Cyclophosphamide Bendamustine Prednisone Hydrocortisone Ibrutinib	Notch1,2,4	GSI-XII Combination of anti-Notch1,2,4 antibodies	Resistance to apoptosis; BMSC-induced glycolytic switch	c-IAP 2 Bcl-2 NF-κB PARP c-myc cyclin b1	Nwabo Kamdje et al., 2012; Jitschin et al., 2015; Secchiero et al., 2017
B-ALL	Hydrocortisone	Notch3,4	GSI-XII anti-Notch3,4 Jagged1,2 and Dll4 antibodies	Resistance to apoptosis	Bcl-2	Nwabo Kamdje et al., 2011
AML	Cytarabine Idarubicin Etoposide	Unknown	GSI-IX GSI-XII	Resistance to apoptosis	STAT3, AKT NF-κB	Takam Kamga et al., 2016
CML	Imatinib	Notch1 Notch2 HES1	GSI-953	↑LSC	PI3K-Akt/mTOR	Aljedai et al., 2015
MM	Doxorubicin Mitoxantrone Bortezomib Melphalan Lenalidomide	Jagged1 Jagged2	GSI-XII Jagged1/2 siRNAs	Resistance to apoptosis; ↓ BM support ↑CSC	p21 NOXA Bcl2 Survivin ABCC1 CXCR4 CYP1A1	Nefedova et al., 2004, 2008; Chiron et al., 2012; Xu et al., 2012a; Garavelli et al., 2017; Muguruma et al., 2017
Osteo-sarcoma	Dox Etoposide Methotrexate CDDP	Dll1 Jagged1	–	Resistance to apoptosis; ↑detoxifying activity; ↑CSC	miR34a-5p ATF2/3/4 miR26a ALDH	Honoki et al., 2010; Mu et al., 2013; Macedo et al., 2017; Pu et al., 2017
Prostate cancer	–	Notch1 Notch4	Notch1 or Notch4 siRNA	↑CSC ↑EMT	NFκB	Zhang L. et al., 2017; Lin et al., 2018
Breast cancer	Lapatinib HT	Notch1 Notch3 Notch4 Jagged1	GSI-IX DBZ Notch1 or Notch4 siRNA	↑CSC ↑bone resorption	IL6/STAT3/oncomiR221	Harrison et al., 2010b; Sansone et al., 2016; Zheng et al., 2017; Shah et al., 2018
NSCLC	Cisplatinum	Notch1	RO4929097	↑CSC hypoxia	STAT3 AKT Survivin	Zhang L. et al., 2017


## Author Contributions

RC and AB conceived, designed, and drafted the manuscript. MC, NP, and DG wrote sections of the manuscript. MTP drew the figures and wrote figure legends. All authors contributed to manuscript revision, read and approved the submitted version.

## Conflict of Interest Statement

The authors declare that the research was conducted in the absence of any commercial or financial relationships that could be construed as a potential conflict of interest.

## Abbreviations

ALDH: aldehyde dehydrogenase
ALDOA: aldolase A
AML: acute myeloid leukemia
B-ALL: B-cell acute lymphoblastic leukemia
BM: bone marrow
BMSCs: bone marrow stromal cells
CLL: chronic lymphocytic leukemia
CML: chronic myeloid leukemia
CSCs: cancer stem cells
EMT: epithelial-mesenchymal transition
ENO1,2: enolase1,2
GSI-XII: gamma-secretase inhibitor XII
HK2: hexokinase 2
ICN: intracellular portion of Notch
LDHA: lactate dehydrogenase A
LSCs: leukemic stem cells
MGUS: monoclonal gammopathy of uncertain significance
MM: multiple myeloma
NSCLs: non-small cell lung cancer
OBL: osteoblast
OS: osteosarcoma
OXPHOS: oxidative phosphorylation
PDK2: pyruvate dehydrogenase kinase 2
PFKM: phosphofructokinase muscle type
ROS: reactive oxygen species
T-ALL: T-cell acute lymphoblastic leukemia
TCA: tricarboxylic acid
